# Account of Diseases in an Eastern District of London, from September 20, to October 20, 1806

**Published:** 1806-11

**Authors:** 


### *Account of Diseases in an Eastern District of London, from September* 20, *to October* 20, 1806.

| | | | |
|---|---|---|---|
| ACUTE DISEASES. | | Variola | 1 |
| Typhus | 3 | Ophthalmia | 2 |
| Peripneumonia | 5 | Rheumatismus Acutus | 3 |

CHRONIC

CHRONIC DISEASES.

| | |
|---|---|
| Tussis . . . . . . . . | 15 |
| Catarrhus . . . . . . | 6 |
| Dyspnœa . . . . . . | 5 |
| Tussis cum Dyspnœa . | 7 |
| Pleurodyne . . . . . | 3 |
| Gastrodynia . . . . . | 5 |
| Enterodynia . . . . . | 9 |
| Diarrhœa. . . . . . | 20 |
| Pyrosis . . . . . . . . | 1 |
| Hæmorrhois . . . . . | 4 |
| Fluor Albus . . . . . | 5 |
| Vertigo . . . . . . | 2 |
| Cephalalgia . . . . . | 7 |
| Chlorosis . . . . . . | 5 |
| Menorrhagia . . . . | 4 |
| Amenorrhœa . . . . . . | 7 |
| Rheumatismus Chronicus | 13 |

PUERPERAL DISEASES.

| | |
|---|---|
| Menorrhagia Lochialis | 4 |
| Ephemera . . . . . | 5 |
| Mastodynia . . . . . | 7 |

INFANTILE DISEASES.

| | |
|---|---|
| Diarrhœa. . . . . . . . | 7 |
| Pertussis . . . . . . . | 3 |
| Pneumonia . . . . . | 2 |
| Convulsio . . . . . . . | 3 |

Those diseases which have their seat in the intestinal canal, and which were mentioned in the last Report, still continue to form a large part of the list. Besides that species of diarrhœa which was then mentioned, and which amounted only to a salutary evacuation of the bowels, without pain or other disease attending it, there have been some instances, in which there was a considerable degree of pain. In these cases, the use of opiates may be admitted under proper regulations, nor is the admission of them at all inconsistent with the caution given in connection with the last report. They are to be administered not with a view to prevent the evacuation, but rather to remove pain, and facilitate the passage of fæces through the intestines; and thus to co-operate with any cathartic remedy which may be employed. A few grains of pulv. ipecac. comp. taken at bed-time, have relieved pain, and produced a gentle diaphoresis, and when this has been followed the next morning by an active cathartic, the most salutary effects have been produced.

